# Parental Perceptions and Decision-Making Processes Regarding Expanded Newborn Screening: A Qualitative Study

**DOI:** 10.3390/ijns12040082

**Published:** 2026-10-05

**Authors:** Masashi Harazaki, Yuka Wada, Mami Notake, Naoki Takabayashi, Fumi Kurebayashi, Philip Hawke, Taichi Hatta, Takeshi Usui, Hiroshi Yamazaki

**Affiliations:** 1 Department of Pediatrics, Shizuoka General Hospital, Shizuoka 420-8527, Japan; 2 Department of Medical Genetics, Shizuoka General Hospital, Shizuoka 420-8527, Japan; yuka-wada@i.shizuoka-pho.jp (Y.W.); mnotake@ntmc-genet.jp (M.N.); n-takabayashi@i.shizuoka-pho.jp (N.T.); yfumi1109@gmail.com (F.K.); tusui1220@gmail.com (T.U.); 3 Graduate School of Public Health, Shizuoka Graduate University of Public Health, Shizuoka 420-0881, Japan; thatta@s-sph.ac.jp (T.H.); hyamazaki@l.u-tokyo.ac.jp (H.Y.); 4 School of Pharmaceutical Sciences, University of Shizuoka, Shizuoka 422-8526, Japan; hawke@u-shizuoka-ken.ac.jp

**Keywords:** newborn screening, expanded newborn screening, parental decision-making, qualitative research, reflexive thematic analysis, informed consent, health communication, rare diseases

## Abstract

Newborn bloodspot screening (NBS) has expanded in scope owing to technological advances and the development of novel therapies. In Japan, coverage of standard NBS is nearly 100%; however, uptake of expanded newborn bloodspot screening (eNBS), which has been increasingly introduced across many regions of the country in recent years, varies across municipalities and healthcare institutions. To explore parental perspectives on eNBS, we conducted a qualitative study using semi-structured interviews with mothers of newborns delivered at Shizuoka General Hospital. Data were analyzed using reflexive thematic analysis. Stratified purposive sampling based on eNBS uptake was used to recruit both parents who accepted and those who declined eNBS, resulting in 24 participants. Three main themes were identified: (1) difficulty understanding unfamiliar rare diseases; (2) subjective interpretation based on personal experiences; and (3) perceptions of screening programs and associated costs. These themes suggested a sequential interpretive process; however, interpretations of the latter two themes varied between participants who accepted and those who declined eNBS. To support informed parental decision-making regarding eNBS and reduce barriers to screening among those who accept it, efforts should include communication tailored to parents’ information needs, measures to prevent social disadvantage and discrimination associated with early identification of disease, and consideration of public financial support.

## 1. Introduction

Newborn bloodspot screening (NBS) is a cornerstone public health program that enables the early detection of congenital or inherited conditions that are often asymptomatic at birth but can lead to serious morbidity or death if left untreated. Early detection allows timely intervention to prevent disability or reduce disease-related harm [1]. Advances in technology, improved clinical understanding, and therapeutic progress have expanded the range of conditions included in NBS programs [2,3]. Although this highly effective preventive strategy is well recognized within the medical community, its value is not always fully appreciated by policymakers, and the implementation of NBS varies substantially across countries depending on national policies [2,4,5].

In Japan, NBS was initiated in 1977 as a national program targeting amino acid metabolism disorders—phenylketonuria, homocystinuria, maple syrup urine disease, and histidinemia—and galactosemia. Histidinemia was discontinued in 1993; congenital hypothyroidism was added in 1979, followed by congenital adrenal hyperplasia in 1989. In 2014, tandem mass spectrometry was introduced nationwide, replacing the traditional Guthrie test. This expansion enabled the inclusion of urea cycle disorders, organic acid metabolism disorders, and fatty acid oxidation disorders, increasing the number of screened conditions to approximately 20 [6].

In recent years, therapeutic advances and improvements in screening technologies have expanded the range of conditions targeted by NBS beyond those included in Japan’s existing standard newborn bloodspot screening (stdNBS) program. To date, expanded newborn bloodspot screening (eNBS) targeting spinal muscular atrophy (SMA) [7], severe combined immunodeficiency (SCID) [8], and selected lysosomal storage diseases [9] has been implemented in Japan at regional or institutional levels, often as a voluntary screening program.

If left undiagnosed and untreated, these conditions can lead to serious complications or death. However, diagnosis at an early or presymptomatic stage enables timely intervention and may improve clinical outcomes [10,11,12,13,14]. At the same time, incorporating rare and generally unfamiliar conditions into screening programs raises important challenges regarding how information should be communicated to parents and how they make decisions about screening [15].

Consent policies for NBS vary internationally and include mandatory screening, in which the government determines whether screening is performed; opt-out models, in which screening is recommended but parents can refuse; and voluntary informed consent, in which screening is recommended but parental consent is required before screening is performed [16,17,18]. In Japan, NBS is conducted under a voluntary informed consent framework, with parental consent being obtained through healthcare institutions. Although the nationwide coverage of stdNBS is nearly 100% [19], the uptake of eNBS varies across municipalities and healthcare institutions [20,21].

In Shizuoka Prefecture, Japan, an eNBS program was initiated in 2023 and currently screens approximately 10,000 newborns annually. The program includes SMA, SCID, B-cell immunodeficiency, mucopolysaccharidosis type I and II, Pompe disease, and Fabry disease (in male newborns only). Approximately 40–50 newborns per year require confirmatory testing, and around two cases are confirmed annually [21]. Standard NBS is provided free of charge and has achieved near-universal coverage. In contrast, eNBS is an optional test that cost approximately 10,000 yen (≅US$70 in 2026) per newborn until April 2025, with an uptake rate of about 80% across the prefecture [21] and 83–84% at Shizuoka General Hospital, where our study was conducted. During the study, Shizuoka Prefecture participated in a national government pilot program for infants born on or after 1 May 2025; consequently, screening for SMA and SCID is currently provided free of charge, whereas testing for the remaining diseases costs approximately 5000 yen (≅US$35) per newborn.

Understanding parental perceptions of eNBS may provide important insights into how information about the screening system should be provided and how parents can be supported in decision-making, thereby enhancing acceptability. Although recent studies have examined the experiences of parents of newborns with positive or false-positive NBS results [22,23,24,25,26,27,28,29], relatively few studies have focused on the decision-making of parents after they receive NBS-related information [18,30,31,32]. Therefore, this study aimed to elucidate the factors influencing parental decisions regarding eNBS uptake and to develop a conceptual framework of the decision-making process. Based on these findings, we further considered how the delivery and content of eNBS-related information, as well as support for parents, could be improved.

## 2. Materials and Methods

### 2.1. Participants

This study included mothers of newborns delivered at Shizuoka General Hospital between December 2024 and June 2025. Information about eNBS was provided by midwives during outpatient prenatal checkups at approximately 32–33 weeks of gestation. Two midwives were primarily responsible for this explanation, which was standardized within the team. Parents who chose to accept eNBS were instructed to submit a consent form to the ward staff on the day of delivery or the following day. Stratified purposive sampling was employed [33,34,35], with priority given to mothers who declined eNBS to ensure adequate representation, aiming for approximately 10 participants in each stratum. The exclusion criteria were as follows: mothers of newborns in critical condition; mothers whose physicians determined that participation in an interview was inappropriate because of their physical or psychological postpartum condition; non-Japanese-speaking mothers for whom participation in a semi-structured interview conducted in Japanese would be difficult; and mothers who declined to participate or declined audio recording.

### 2.2. Data Collection

Participants were provided with detailed information about the study, and written informed consent was obtained either on the day before or on the day of the interview. To minimize potential pressure on mothers who had declined eNBS regarding their screening decision, interviews were conducted by genetic counselors who had no direct involvement in the participants’ clinical care or screening decisions. Semi-structured interviews were conducted by trained genetic counselors using an interview guide (see Appendix A) on postpartum days 2–4. Face-to-face interviews were conducted either in a consultation room on the ward or in the participant’s private hospital room. With participants’ consent, the interviews were audio-recorded and subsequently transcribed verbatim. During transcription, names and other identifiable personal information were replaced with pseudonyms to protect participants’ privacy and ensure confidentiality. Data analysis was conducted using these transcripts.

Following discharge, mothers and their infants returned for a routine one-month checkup, during which a pediatrician provided the eNBS results. The genetic counselor subsequently met directly with participating mothers to confirm whether they had any additional comments or corrections regarding their previous responses. As compensation, participants received a 1000-yen (≅US$7) gift card after the one-month checkup.

### 2.3. Data Analysis

Data were analyzed using Braun and Clarke’s reflexive thematic analysis method [36]. This method was used because it supports the development of themes not merely as summaries of data patterns, but as interpretive constructs through which a nuanced understanding of participants’ experiences can be developed. 

Ontologically, we adopted a realist position, assuming that social, institutional, and cultural realities are not reducible to participants’ perceptions or researchers’ interpretations [37,38]. Epistemologically, we regarded knowledge of these realities as necessarily partial, situated, and fallible, as it is shaped by participants’ and researchers’ experiences, sociohistorical contexts, and analytical perspectives [37,39]. The analysis was primarily inductive and interpretive, with an emphasis on understanding participants’ experiences and meaning-making. At the same time, participants’ accounts were interpreted in relation to the broader social, institutional, and cultural contexts in which their perceptions and meanings were formed [40].

The analysis began with familiarization with data through repeated listening to audio recordings and careful reading of transcripts. Coding was conducted inductively at both semantic and latent levels without a predefined coding framework. The final themes were primarily developed from semantic codes to remain closely grounded in participants’ explicitly described experiences and concrete circumstances, while latent coding was used where appropriate to explore underlying meanings and assumptions. Data were interpreted empathetically, with an emphasis on understanding participants’ experiences. Initial candidate themes were developed iteratively with the aid of visual mapping and were further refined by comparing them with the coded data and the entire dataset. The final themes were then defined and named through this process. The analysis was iterative and recursive, involving ongoing review and refinement of codes and themes throughout the analytical process. Analytical rigor was supported by critically evaluating the analysis against established quality criteria for reflexive thematic analysis, including internal coherence, distinctiveness, and a central organizing concept [41]. Research practices were further guided by the Reflexive Thematic Analysis Reporting Guidelines (RTARG) [42]. Data management and analysis were carried out with NVivo 15 software, 2024 release (Lumivero, Denver, CO, USA), together with manual analysis.

### 2.4. Reflexivity and Research Quality

Reflexivity was integrated throughout the study design, data collection, and analysis to enhance analytical rigor and trustworthiness. MH is a pediatrician and clinical geneticist with clinical experience in neonatal care and in managing children with SMA who have severe motor impairment and their families. Throughout the study, MH remained aware that his clinical background and the value he places on early detection and intervention for conditions targeted by eNBS could influence his interpretation of the data. 

The interviewers, YW, MN, NT, and FK, are genetic counselors and therefore brought professional perspectives as healthcare practitioners that could have influenced the interview process. From the outset of data collection, they explicitly sought to maintain a non-directive stance toward participants’ decision regarding whether to undergo eNBS and did not privilege either acceptance or decline of screening. The purpose of the interviews was not to encourage a particular choice, but to explore how participants understood eNBS and how their perceptions shaped their decision-making. When participants expressed misunderstandings or inaccurate perceptions about eNBS, the interviewers did not immediately correct them during the interview so that the participants’ understanding at the time of decision-making could be explored. After the interview had concluded, however, the genetic counselor provided accurate information as appropriate.

To support reflexive consideration of the interview process and elicit participants’ perceptions in greater depth, YW, MN, NT, and FK discussed interviewing approaches with MH before the interviews and, after each interview, discussed the content of the interview, including the way questions had been asked and interactions with participants. 

Although reflexive thematic analysis does not require multiple analysts, MH discussed the analysis throughout the analytic process with HY and TH to facilitate reflexive and critical dialogue. MH is a pediatrician and clinical geneticist, whereas TH and HY are qualitative researchers who are not healthcare professionals. The three analysts considered how their differing professional backgrounds, assumptions about screening, and interpretive perspectives might shape the interpretation and coding of the data. These reflexive and critical discussions were used to make the analysts’ assumptions and interpretive positions more explicit and contributed to the iterative refinement of the themes.

Among the other authors, TU is a physician who contributed primarily to study resources, while PH, who is not a healthcare professional, contributed primarily to supervision and manuscript development. Neither was directly involved in the interviews, coding, or theme development. 

## 3. Results

### 3.1. Participant Characteristics

A total of 26 mothers were approached; 24 participated in the study and were interviewed. The participants’ characteristics are summarized in Table 1. Of the 24 participants, four gave birth on or after 1 May 2025, and were enrolled in the government pilot program; therefore, their out-of-pocket expense for eNBS was reduced by half. Of these four participants, two declined the fee-based components of eNBS. At the time of the interview, the clinical status of the newborns was as follows: 22 were healthy, one had low birth weight, and one had neonatal jaundice. No newborns had positive or false-positive eNBS results. Interview durations ranged from 14 min 20 s to 50 min 20 s, with a median of 24 min 37 s.

### 3.2. Qualitative Findings

**Figure 1:**
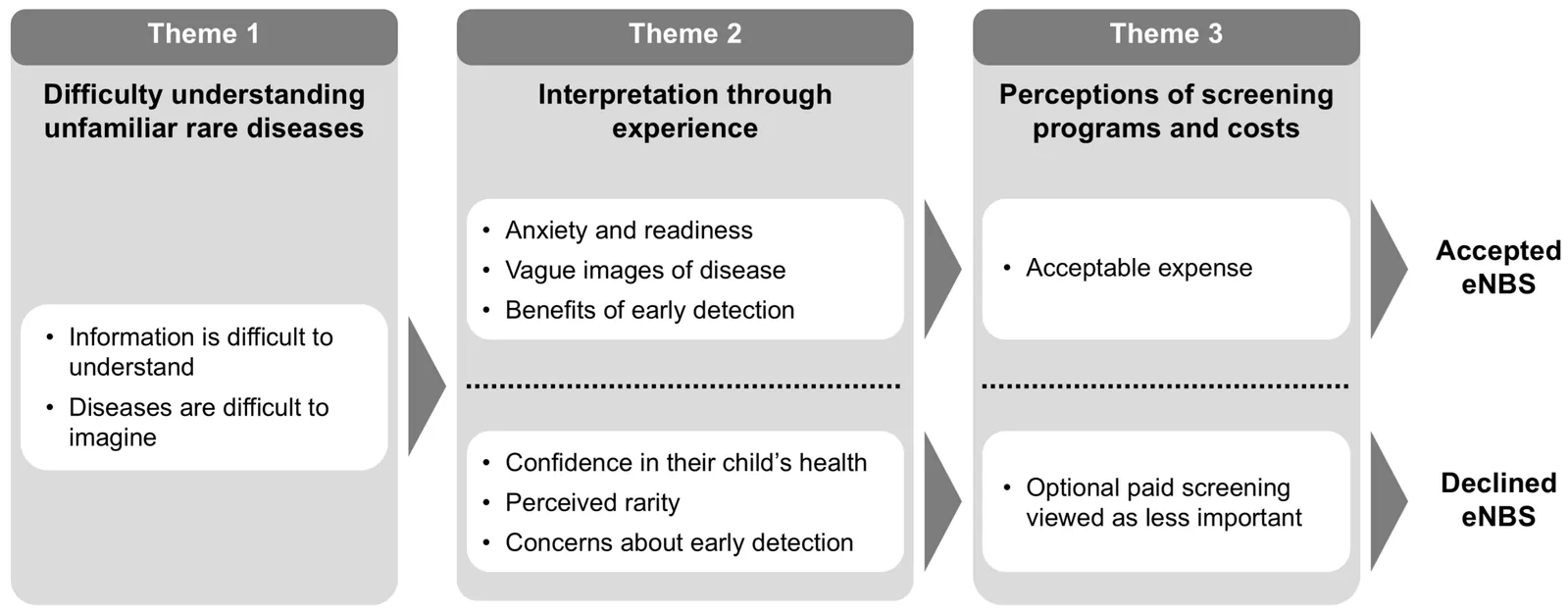
Conceptual framework illustrating the parental decision-making process regarding expanded newborn bloodspot screening (eNBS). Parents first encounter information about unfamiliar rare diseases that is difficult to understand (Theme 1). They then interpret this information through the lens of their personal experiences (Theme 2), and this interpretation shapes their perceptions of screening programs and their associated costs (Theme 3). These themes are interconnected and can be understood as a sequential interpretive process.

Three main themes and ten subthemes were identified. The main themes were as follows: (1) difficulty understanding unfamiliar rare diseases; (2) subjective interpretation of screening information based on personal experiences; and (3) perceptions of screening programs and associated costs (Figure 1). Parents were confronted with screening decisions regarding rare diseases with which they were largely unfamiliar. Although the information provided was difficult for them to fully understand, parents interpreted it through the lens of their own experiences and developed their own value judgments about the two screening programs and associated costs. These findings suggest that the three themes can be understood as a sequential interpretive process, generally progressing from Theme 1 to Theme 3. Detailed descriptions of each theme and its subthemes are provided below.

### 3.3. Theme 1: Difficulty Understanding Unfamiliar Rare Diseases

Difficulty understanding the conditions targeted by eNBS was similarly evident among participants who accepted eNBS and those who declined it. Narratives related to this theme were elicited primarily when participants were asked, in accordance with the interview guide, to reflect on the explanations and informational materials they had received about eNBS, rather than emerging as concerns that participants spontaneously and repeatedly emphasized. Accordingly, these difficulties in understanding were interpreted not as factors that directly differentiated decisions to accept or decline eNBS, but rather as a common background to decision-making in both groups.

Participants’ accounts indicated two interrelated aspects of this difficulty. One was the challenge of adequately understanding information about eNBS when it was provided in late pregnancy alongside a substantial amount of other information. The other was participants’ lack of familiarity with the rare conditions included in eNBS, which made it difficult for them to form a concrete picture of these conditions.

#### 3.3.1. Difficult-to-Understand Information

Information about eNBS was typically provided by midwives during prenatal checkups at approximately 32–33 weeks of gestation, together with explanations of hospital admission procedures and childbirth preparation. When participants were asked during the interviews to reflect on how this information had been provided, both those who accepted eNBS and those who declined it described difficulty paying sufficient attention to the content of eNBS and remembering its details while receiving a large amount of information at the same time. For example, Mother 1, who accepted eNBS, recalled that the eNBS materials had been included among the many documents she received during pregnancy and that she took them home without fully understanding their contents.

*“It was just one of many hospital admission documents, like the maternity life guide, that were all given to me at once, so I took it home without really understanding it.”*

(Mother 1, accepted eNBS)

In both groups, some participants also described difficulty understanding due not only to the amount of information provided but also to the explanations themselves, which relied largely on disease names and written text. In particular, for participants with little or no prior knowledge of the target conditions, disease names alone were insufficient to help them form a concrete understanding of the characteristics of the diseases or the significance of screening. Mother 2, who declined eNBS, reflected that illustrations or simpler explanations that would help her visualize the conditions might have made them easier to understand.

*“Even if they list the name of the disease, I don’t really understand it. If there were pictures or something—like showing what the disease looks like—something beyond just text, something easier for people like us to understand, then I might think, ‘Oh, I see.’“*

(Mother 2, declined eNBS)

Thus, difficulty understanding the information was common both to participants who accepted eNBS and to those who declined it, but this difficulty itself did not appear to be directly associated with the decision to decline eNBS.

#### 3.3.2. Difficult-to-Imagine Diseases

Lack of familiarity with the target condition was common to both participants who accepted eNBS and those who declined it. When participants were asked during the interviews to reflect on their understanding of the target conditions, they described both having rarely, if ever, heard the disease names and finding it difficult to imagine the severity or specific symptoms of the conditions from the names alone. 

For example, Mother 4, who declined eNBS, recalled that many of the disease names listed in the materials were unfamiliar to her and that the names alone did not help her understand what kind of conditions they were.

*“There were lots of disease names listed, but they were mostly names I have never heard before, […] so nothing really stood out to me, and I couldn’t understand what made them serious or frightening diseases.”*

(Mother 4, declined eNBS)

Similarly, Mother 8, who accepted eNBS, also described being unfamiliar with the names of target conditions and said that, even after receiving an explanation, the information did not leave a strong impression on her.

*“The disease names were pretty unfamiliar to me, so I just thought, ‘Oh, so you can screen for things like this.’ But honestly, if you asked me now, I probably wouldn’t remember any of them.”*

(Mother 8, accepted eNBS)

With this limited understanding, participants drew on their own past experiences and events in their immediate surroundings as points of reference for interpreting the target conditions and the meaning of screening. Differences in these interpretations, in turn, contributed to differences in decision-making, as described in the following theme.

### 3.4. Theme 2: Subjective Interpretation of Screening Information Based on Personal Experiences

How participants made sense of information about these unfamiliar rare conditions differed according to their own prior experiences. Experiences such as anxiety surrounding pregnancy and childbirth, concerns or experiences related to their children’s health, consideration of prenatal testing, and illnesses or developmental problems among people close to them served as points of reference through which participants interpreted unfamiliar conditions in relation to their circumstances. Unlike the difficulties described in Theme 1, differences in these interpretations were also associated with participants’ decisions about whether to accept eNBS.

#### 3.4.1. Anxiety About Diseases and Readiness to Accept a Potential Diagnosis in Their Children

The extent to which participants perceived unexpected health problems as something that could realistically occur in their own child varied according to their previous experiences of pregnancy, childbirth, and their children’s health. Among participants who accepted eNBS, more than half spontaneously described concerns about their child’s health. For some, these concerns had begun during pregnancy and had not completely resolved after delivery. They viewed eNBS as a means of reducing, at least to some extent, uncertainty about the future.

Mother 1 reflected on the anxiety she had experienced during pregnancy about whether her baby would be born healthy and explained that, even after a safe delivery, she would likely have continued to feel anxious if she had chosen not to have her newborn screened.

*“Before giving birth, I was worried about whether the baby would be born healthy. If I hadn’t had the test, I think I would have kept worrying.”*

(Mother 1, accepted eNBS)

These perceptions of their child’s health were not necessarily fixed and could change in response to events after birth. Mother 7 had initially decided against eNBS, but after seeing her newborn undergo treatment for neonatal jaundice, she came to perceive the possibility of disease affecting her child as more immediate, and subsequently changed her decision in favor of eNBS.

*“He developed jaundice and had to go into an incubator. Seeing him like that was really hard. So we thought, ‘If something like this can happen, maybe we should just go ahead and have the test done in advance.’”*

(Mother 7, initially declined and later accepted eNBS)

For some participants, their own age and anxiety about becoming a parent for the first time also contributed to a heightened awareness of uncertainty regarding their child’s health and future parenting. Mother 14, who was of an advanced maternal age and giving birth for the first time, described not only worrying about whether her child would be born healthy but also wondering whether she would be able to manage parenting in the same way as those around her.

*“I kept wondering whether the baby would be born healthy, and since I got pregnant later than my friends, I was always thinking about whether I’d be able to go through the same things they had.”*

(Mother 14, accepted eNBS)

Experiences of considering the possibility that their child might have a disease or disability were also reflected in the accounts of participants who had considered prenatal testing, including non-invasive testing. Among those who had considered prenatal testing but ultimately decided not to undergo it, some had already thought during that decision-making process about the possibility that their child might have a health problem and about how they would accept and respond to such a possibility as parents. Therefore, deciding not to undergo prenatal testing was not necessarily inconsistent with subsequently choosing eNBS.

*“My husband and I talked about [prenatal testing] and decided that, once we had chosen to have a child, it would be our responsibility to accept whatever might happen. That’s why we decided not to have prenatal testing.”*

(Mother 16, accepted eNBS)

Thus, among participants who accepted eNBS, some appeared to have chosen screening not simply because they had greater anxiety about disease, but also because, through their experiences of pregnancy, childbirth, and considering prenatal testing, they had already come to view the possibility that their child might have a health problem as something personally relevant to them. These experiences provided a context in which they did not regard the possibility of the unfamiliar conditions targeted by eNBS as entirely unrelated to themselves and were able to consider the value of identifying such conditions early.

#### 3.4.2. Vague Mental Images of Unfamiliar Diseases

Regardless of whether they accepted or declined eNBS, participants generally had limited knowledge of the conditions targeted by the screening. However, several participants who accepted eNBS drew on their understanding of diseases that they already knew or on the health- and development-related experiences of people close to them when trying to make sense of these conditions. Although such experiences did not provide an accurate medical understanding of the conditions included in eNBS, they served as points of reference that helped these participants form their own more concrete impressions of unfamiliar diseases and consider the need for screening.

For example, although the condition was unrelated to those targeted by eNBS, Mother 8 was familiar with the experience of a friend’s child who had shown behavioral difficulties participating in group settings from early childhood and later underwent specialist evaluation. Through this experience, she had come to see value in identifying a child’s problems at an early stage.

*“A friend’s child has had difficulty sitting still since kindergarten and struggled to fit in with groups. That has continued, and even after starting school, the child has had difficulty keeping up with lessons. Hearing that the child is now seeing a specialist and undergoing various tests makes me think that kind of screening is important.”*

(Mother 8, accepted eNBS)

When participants found it difficult to understand unfamiliar rare conditions directly, they supplemented their understanding by drawing in more familiar health-related experiences they had encountered. However, these analogies were not necessarily based on the medical characteristics of the target conditions; rather, they functioned as subjective frames through which participants formed their perceptions of screening.

#### 3.4.3. Expectations Regarding Early Detection

The value of early detection was recognized to some extent not only by participants who accepted eNBS but also by those who declined it. However, the two groups differed in the importance they attributed to its benefits and in how they understood the need to identify these conditions early.

Participants who accepted eNBS regarded the possibility that some of the target conditions were treatable and that early detection and treatment could improve their child’s prognosis as important reasons for choosing screening. Mother 8 considered early detection, before symptom onset or at an early stage of disease, to be particularly valuable because some of the target conditions were treatable.

*“I think it’s because there are treatments available. If the condition can be found early and treated, that definitely matters.”*

(Mother 8, accepted eNBS)

Some participants who accepted eNBS also perceived benefits of early detection beyond its potential impact on treatment outcomes. They valued knowing in advance that their child had a condition because this could allow them to prepare psychologically and to better understand and respond to their child’s growth and symptoms. Mother 19 felt that knowing the diagnosis might help her understand the reasons for any future concerns about her child’s development or growth and give her greater psychological readiness to cope with them.

*“As your child grows, you start to wonder, ‘Why isn’t my child developing like others?’ But if you understand the reasons, you might think, ‘Okay, maybe that’s just how it is,’ and that can give you some peace of mind.”*

(Mother 19, accepted eNBS)

Participants who declined eNBS, however, did not necessarily see no value in early detection. For example, Mother 9 recognized that she might regret not having chosen eNBS if she later found out that the condition could have been identified earlier through screening.

*“I suppose that if my child developed that condition later on, I might regret not having screening done earlier and think we could have found it sooner.”*

(Mother 9, declined eNBS)

However, among some participants who declined eNBS, the distinction between identifying a condition early through screening and diagnosing it after symptoms had developed was not fully recognized. Mother 2 believed that if her child had one of these conditions, symptoms would eventually appear, and therefore did not feel a strong need for screening before symptoms emerged.

*“I believe that if my child has a disease, symptoms will show up anyway. So, I don’t really see the need to check it early, and that’s why I’m hesitant.”*

(Mother 2, declined eNBS)

Rather, differences in how these participants valued eNBS were related to whether they could envision concrete benefits of early detection for treatment, prognosis, and psychological preparedness.

#### 3.4.4. Confidence in Their Child’s Health

More than half of the participants who declined eNBS drew on their previous parenting experiences as a reference point for judging the health of their newborn. In particular, having an older child who had grown up healthy without undergoing eNBS contributed to a sense of reassurance that their current child was also unlikely to have a serious condition. Rather than serving as a basis for evaluating the medical necessity of eNBS itself, these past experiences provided a framework through which participants interpreted the risk of unfamiliar conditions in light of their own family experiences.

For example, Mother 6 cited the fact that her older child had not been offered eNBS at birth and had nevertheless grown up healthy as one reason she felt that eNBS was not necessary for her currently eligible child.

*“When my older daughter was born, this test was not available yet, so we didn’t have it done. She has grown up perfectly healthy, so I feel like maybe my younger daughter doesn’t need to have it either.”*

(Mother 6, declined eNBS)

Although having previously raised a healthy child did not guarantee the health of a subsequent child, for some participants who declined eNBS, this experience provided a basis for perceiving the likelihood of disease in their currently eligible child as low.

#### 3.4.5. Perceived Rarity of the Diseases

Participants’ interpretations that the target conditions were unlikely to affect their own child were also shaped by their limited exposure to these diseases in everyday life. The informational leaflet about eNBS did not provide data on the incidence of the target conditions, and several participants who declined eNBS used their own experiences—such as never having heard of the conditions or not knowing any children with such diseases—as cues that the likelihood of their own child being affected was low. Mother 9 similarly reflected that, because she did not know any children with congenital conditions, she had rather casually assumed that her own child would probably be fine.

This pattern was also clearly evident in Mother 24’s account. Although she recognized that she did not know the actual incidence of the target conditions, she inferred that the likelihood of her own child being affected was low because she had never heard of the diseases and did not know anyone around her who had such conditions.

*“I don’t know the exact numbers for these conditions—like how common they are—but since I’ve never heard of them and no one around me has mentioned anything like that, I just figured the chances were probably low.”*

(Mother 24, declined eNBS)

As such, participants who declined eNBS tended to infer low disease likelihood from their lack of personal exposure to these conditions, which in turn reduced their perceived need for screening.

#### 3.4.6. Concerns About Early Detection

Some participants who declined eNBS did not reject the benefits of early detection but perceived potential disadvantages in finding out early that their child had a condition. Such accounts were rarely expressed by participants who accepted eNBS. These concerns varied according to participants’ own past experiences and how they envisioned life following a diagnosis.

Mother 17 spontaneously expressed concern that early identification of a condition in her child might disadvantage the child when applying for life insurance if the condition was related to a genetic abnormality. In this case, her cautious attitude toward eNBS did not arise from a lack of understanding of the value of early diagnosis. Rather, it reflected how she applied her own experience of social disadvantage following a diagnosis to her expectations about her child’s future.

*“The information mentioned genetic conditions. I have a genetic condition myself, so I was worried that if it became clear now, my son might have trouble getting insurance. That would be a serious concern for us.”*

(Mother 17, declined eNBS)

In contrast, Mother 24’s concern was directed less toward financial or institutional disadvantages and more toward the possibility that knowing about the condition could change how she perceived and experienced life with her child thereafter. She felt that, if a condition were identified soon after birth, the child might go through subsequent life constantly conscious of the illness, creating a greater psychological burden than if she had lived without knowing about it.

*“If a condition is detected right after birth, the child would have to live with it for their whole life. That’s not the same as not knowing. It would mean growing up with that awareness from the start, and I think that could be a lot more stressful.”*

(Mother 24, declined eNBS)

For some participants, anticipated social or psychological burden of an early diagnosis outweighed its perceived benefits and contributed to their decision to decline eNBS.

### 3.5. Theme 3: Perceptions of Screening Programs and Associated Costs

The cost of eNBS was relevant to both participants who accepted eNBS and those who declined it, but the meaning of this cost differed between the two groups. Participants evaluated the cost not in isolation, but in relation to the perceived benefits of screening and to how eNBS was positioned within the healthcare system. 

#### 3.5.1. Perceiving eNBS as an Acceptable Expense

Participants who accepted eNBS did not necessarily consider the screening inexpensive. However, when they believed that early detection and treatment might help prevent adverse health consequences in the future, they regarded the cost as worth paying. In other words, the acceptability of the cost was judged not only by the amount itself but also in relation to the perceived benefit of screening.

Mother 10 recognized that a cost of approximately 10,000 yen (≅US$70) was high but considered it worth paying because she was concerned that not undergoing screening could delay the detection and treatment of a condition.

*“The test costs about 10,000 yen, which certainly feels expensive. But at the same time, that amount could affect the child’s future—well, maybe not completely, but if a condition is found early and treated, things could turn out much better. If you don’t spend that money and only find out later, it might lead to long-term problems. When I think about it like that, I feel it’s probably better to have the test.”*

(Mother 10, accepted eNBS)

Some participants also evaluated the cost of eNBS not in isolation but in comparison with other out-of-pocket healthcare options they considered in relation to pregnancy and childbirth. In these comparisons, prior consideration of more expensive options, such as prenatal testing or respiratory syncytial virus (RSV) vaccination during pregnancy, provided a reference point that made the cost of eNBS seem relatively more acceptable.

Mother 19 reflected on the cost of a vaccine she had considered during pregnancy and the uncertainty she perceived regarding its effectiveness. By comparison, she felt that eNBS, which could identify a disease, would still be worth considering even at a relatively higher cost.

*“[The vaccine] is not a test, but [about the vaccine I thought] ‘Even if you get vaccinated, you can still get the disease, right?’ They said it takes about two weeks after vaccination for antibodies to pass from the mother to the baby. So if the baby is born during that time, it doesn’t really help. That made it feel like a bit of a gamble, so it seemed expensive. But with this test, I can be sure of the results, and even if it had cost 30,000 yen, I feel like it’s something worth considering.”*

(Mother 19, accepted eNBS)

Thus, perceiving the cost as high did not necessarily lead participants to decline eNBS when they considered the potential benefits to justify the expense.

#### 3.5.2. Perceived Lower Importance of Optional Paid Screening

By contrast, more than half of the participants who declined eNBS used not only the financial burden itself but also the way the screening was structured—as optional and fee-based—as cues for judging its necessity.

Although stdNBS is conducted on the basis of informed consent, it is provided free of charge and is received by nearly all newborns. Consequently, many participants perceived stdNBS as a screening test that, in practice, all newborns were expected to undergo or that was simply taken for granted as part of routine care. In contrast, the way eNBS was offered—requiring those who wished to undergo screening to pay an additional fee—was interpreted by some participants as suggesting that it was less important than stdNBS.

Mother 2 questioned whether it was necessary to pay an additional fee for the screening and noted that, if no abnormality were ultimately found, she might feel that money had been “wasted.”

*“The fact that it cost extra money made me think, ‘Hmm, is it really worth paying for?’ And if I paid that and it turned out there was nothing wrong, I’d feel like it was such a waste.”*

(Mother 2, declined eNBS)

In Mother 13’s account, however, it was not only the financial burden but also the fact that the screening was optional that influenced how she interpreted its importance. She viewed a screening test received by everyone as more necessary, whereas the system in which only those who wished to undergo eNBS paid for it led her to perceive eNBS as relatively less important.

*“If it had been mandatory instead of optional, I would have thought it must be important. But since it was a choice and cost extra, I felt maybe it wasn’t that important after all.”*

(Mother 13, declined eNBS)

For some participants, the fee-based and optional nature of eNBS jointly signaled lower importance than stdNBS and contributed to their decision to decline screening.

## 4. Discussion

This study revealed that, through comparisons with other screening programs, parents interpreted the importance and necessity of eNBS in ways that influenced their decision-making. Their decisions were shaped not only by the financial burden of this optional screening test but also by the difficulty of understanding unfamiliar rare diseases and by their interpretation of screening information through their own experiences. The findings suggest that parents did not simply accept or decline eNBS based on cost alone; rather, their decisions were shaped through a sequential interpretive process involving information comprehension, personal experiences, and perceptions of screening programs and associated costs.

### 4.1. Providing Information on eNBS and Ensuring Parental Understanding

When providing information about eNBS, communication tailored to parents’ needs is essential. In this study, midwives provided parents with leaflets published by the Shizuoka Prefecture Rare Disease Network, flyers containing information on costs, and links to relevant websites. However, participants often read only selected parts of these materials, and even when they did read them, they did not always fully understand the content. This was mentioned not only by participants who declined eNBS but also by those who accepted it. Participants perceived the leaflets and flyers as text-heavy, which led some to lose interest before reading them in full. They also found the content difficult to understand because many of the disease names were unfamiliar. One participant noted that, whereas disease names such as “spinal muscular atrophy,” which indicate the affected organ or body system, made it relatively easy to imagine the condition, many other disease names did not help them understand what the conditions entailed.

Previous studies have highlighted the need for parental education regarding NBS [32,43,44,45]. It has also been reported that parents who receive insufficient explanations before screening, or who are given incorrect information, may be particularly vulnerable to emotional distress when informed of abnormal NBS results [32,43,44]. Although the present study did not include parents whose newborns required confirmatory testing after stdNBS or eNBS results, participants expressed a need for easily accessible and understandable information. These findings suggest that promoting parental understanding of eNBS requires not only the provision of information but also support tailored to parents’ needs. Such support may include adjusting the amount of information provided at one time; using videos and illustrations in addition to written materials; and, for parents who already have children, providing updated information that reflects advances in medicine and healthcare.

### 4.2. Perceptions of Rare Diseases Influenced by Personal Experiences

Parents’ perceptions of rare diseases were strongly shaped by their personal experiences. Although these diseases are individually rare, newborn screening and epidemiological studies in Japan have reported estimated frequencies of approximately 1 in 7000–8000 newborns for Fabry disease [46] and approximately 1 in 20,000 live births for SMA [47]. Consistent with this rarity, participants generally had little or no exposure to these diseases.

Participants who accepted eNBS tended to form vague images of illness or disability, influenced by parenting-related anxieties, including those arising from a lack of prior parenting experience and those grounded in actual parenting experiences. By contrast, participants who declined eNBS tended to conclude that their newborn was unlikely to be affected by the conditions screened for. This perception appeared to be based on difficulty of imagining unfamiliar rare diseases, not knowing anyone close to them with congenital conditions, and confidence gained from previous parenting experiences.

Previous studies have discussed the influence of parental experience on consent, including findings that some parents perceived blood collection from a newborn’s heel as distressing or even torturous [18,30]. In Japan, postpartum hospital stays are generally longer than in many Western countries. A recent Japanese prospective observational study reported a median time from delivery to discharge of 4.85 days after spontaneous vaginal delivery and 5.99 days after scheduled cesarean delivery [48]. Blood samples for eNBS are often collected during this postpartum hospitalization, but parents do not necessarily witness the procedure firsthand. Furthermore, because the screening rate for stdNBS is nearly 100%, very few parents refuse the blood draw itself [19]. However, high uptake of NBS is not unique to Japan and has been reported in many other countries, including settings where participation is not mandatory [49]. Therefore, the near-universal uptake of stdNBS in Japan should not be attributed solely to the Japanese sociocultural context. Nevertheless, social conformity and group-oriented behavior may contribute to the way some parents perceive participation in screening programs in Japan [50,51,52]. 

A pilot study of newborn screening for the *FMR1* gene in North Carolina, USA, found that parents’ decisions to accept or decline screening were related to how they perceived its potential benefits and drawbacks [53]. In the present study, parents did not simply respond to the information provided; rather, they interpreted the same information differently through the lens of their own experiences, including pregnancy and childbirth, parenting, prenatal testing, and illnesses affecting people close to them. Previous studies have likewise shown that people draw on the experiences of others and on personal narratives when trying to understand unfamiliar health conditions or healthcare choices [54,55]. Taken together, these findings suggest that personal experiences may provide an interpretive framework through which parents make sense of unfamiliar conditions targeted by eNBS and assess the value of screening.

### 4.3. Clinical Benefits and Social Drawbacks of Early Detection

As eNBS facilitates early detection and access to timely treatment, attention should also be paid to potential disadvantages associated with early identification, including issues related to life insurance and discrimination. The primary purpose of NBS is to detect diseases soon after birth and to implement measures to prevent disability or reduce disease-related harm. In this study, participants who accepted eNBS interpreted screening information through the lens of their own experiences. Even when they did not fully understand the specific conditions included in eNBS, they had some awareness of the importance of early detection and treatment. They appeared to recognize the value of screening more readily when they could picture relatively common neurodevelopmental conditions familiar to them, such as autism spectrum disorder and attention-deficit/hyperactivity disorder. They also perceived the need for screening in the light of their desire to avoid a prolonged diagnostic journey and their belief that early detection could enable early intervention. Previous studies have similarly identified the desire to avoid a prolonged diagnostic journey as one reason for having a positive attitude toward eNBS [56,57]. By contrast, although participants who declined eNBS acknowledged that early detection and treatment were desirable, some also identified potential drawbacks of obtaining such information. These included the emotional burden associated with such knowledge, concerns about eligibility for life insurance, and fears of discrimination. Previous research suggests that such concerns reflect broader social and psychological issues associated with early identification [58].

Although many conditions targeted by eNBS have a genetic basis, many countries, particularly in Europe and North America, have adopted various policy approaches to address genetic discrimination [59]. In Japan, the Genome Medicine Promotion Act, officially titled the Act on the Comprehensive and Planned Promotion of Measures to Ensure That the Public Can Receive High-Quality and Appropriate Genome Medical Care with Peace of Mind, came into effect in June 2023. Based on this Act, the Basic Plan for Genome Medical Care, which includes measures to prevent discrimination and establish systems for the provision of genomic medical care, was approved by the Cabinet in November 2025 [60,61]. However, some scholars have argued that concerns about discrimination may persist even after legal protections against genetic discrimination are strengthened [62]. As early disease detection and timely treatment become more widely implemented, it will be important to consider not only the clinical benefits of these approaches but also their potential social drawbacks, including issues related to life insurance eligibility and discrimination.

### 4.4. Individual Cost-Sharing for Public Health Programs

As with stdNBS, public funding may also be warranted for eNBS. In Japan, consent for NBS has traditionally been obtained through voluntary informed consent procedures. However, because stdNBS is provided free of charge and has nearly universal coverage, some participants perceived it as a mandatory screening program. This phenomenon is not unique to Japan. A previous study in the United Kingdom, where NBS is provided within an informed-choice framework, reported that one-third of parents felt that they did not have the option to decline NBS [63]. In other words, some participants appeared to distinguish between publicly funded screening that felt mandatory and optional screening that required out-of-pocket payment. Based on this contrast, some participants judged eNBS to be relatively less important. As noted in a previous study, the goal of increasing screening uptake as a public health program and the goal of promoting informed decision-making may also be ethically in tension [64,65]. Introducing public funding may increase screening uptake, but it may also encourage parents to accept screening without sufficient deliberation, thereby undermining informed decision-making. 

On the other hand, the prolonged coexistence of publicly funded and self-paid screening programs could widen health disparities between socioeconomic groups. Therefore, efforts to support informed parental decision-making regarding eNBS should not only provide parents with appropriate information and communication but also ensure that those who choose screening can act on that decision. This requires consideration of public financial support, as well as adequate systems for screening and treatment.

Relatedly, genome-sequencing research projects involving apparently healthy newborns are underway in some countries [66]. If such initiatives continue to develop, fee-based genomic sequencing for newborns may eventually be offered as an optional service outside the research context. Genomic sequencing-based screening of apparently healthy newborns remains ethically and practically debated, and the broader implementation such screening may raise similar questions to those identified in this study: how parents understand optional screening, weigh potential benefits and drawbacks, and make decisions on behalf of their child.

### 4.5. Strengths and Limitations of This Study

A key strength of this study is its qualitative approach, which enabled an in-depth exploration of diverse parental experiences and perspectives. Rather than focusing on the most common or average experiences, this approach allowed for nuanced interpretations of data reflecting varied individual experiences. It also yielded narratives grounded in participants’ personal contexts, which may be difficult to capture through focus group interviews. This is particularly important in healthcare practice, where families should have access to care tailored to their individual needs and preferences. Another key strength of this study is that it included parents who declined eNBS. Although several studies have explored parental perceptions of NBS, little has been reported about the experiences of parents who decline NBS [18,30]. Because all newborns in this study underwent stdNBS, the participants’ situations differed from those of parents who decline NBS entirely. Nevertheless, parental perceptions of expanded screening as an optional service should be considered an important public health issue.

However, this study also has several limitations. Because it was conducted in a single regional referral hospital where eNBS was introduced under specific organizational conditions, parents’ experiences may differ in other healthcare settings. Although participants were contacted approximately one month after the interviews and invited to provide additional comments or report changes in their views, the findings primarily reflect mothers’ experiences during the immediate postpartum period. Many participants described discussing eNBS with their partners and making decisions jointly as parents; however, fathers’ perspectives were not directly explored. In addition, mothers of newborns with severe medical conditions and parents of newborns referred for confirmatory testing after either stdNBS or eNBS were not included. Consequently, this study does not fully represent the perspectives of fathers, parents whose newborns had severe medical conditions, parents of newborns referred for confirmatory testing after positive stdNBS or eNBS results, nor does it reflect how parents’ views may develop beyond the short follow-up period.

Future research should include multicenter studies in diverse healthcare settings, as well as interviews with fathers and with parents of newborns referred for confirmatory testing. Longitudinal studies may also help clarify how parents’ understanding of eNBS and their reflections on decision-making evolve over time.

## 5. Conclusions

Parents’ personal experiences play an important role in how they interpret information and make decisions regarding eNBS. Supporting informed parental decision-making requires providing information and communicating in ways that are responsive to parents’ needs. For parents who choose eNBS after considering such information, efforts to reduce the social disadvantage and discrimination associated with early disease identification, together with consideration of public financial support, may help reduce barriers to newborn screening.

## Figures and Tables

**Table 1:** Characteristics of interview participants (*n* = 24).

eNBS Decision	Accepted eNBS (*n* = 13)		Declined eNBS (*n* = 11)	
	*n*	%	*n*	%
Age in years				
26–30	6	46	5	45
31–35	5	39	4	36
36–40	2	15	2	18
Highest educational attainment *				
Junior high school	0	0	1	9
High school	4	31	4	36
2-year college/vocational school	2	15	1	9
University (bachelor’s)	7	54	5	45
Graduate school (master’s/doctoral)	0	0	0	0
Number of children, including the newborn				
One	6	46	2	18
Two	7	54	3	27
Three or more	0	0	6	54
Occupation **				
Professional	4	31	3	27
Healthcare	0	—	1	—
Childcare and education	4	—	1	—
Other professional	0	—	1	—
Clerical worker	2	15	2	18
Service or sales worker	4	31	4	36
Skilled manual worker	0	0	0	0
Unskilled laborer	0	0	0	0
Homemaker	3	23	2	18

* Junior high = compulsory; 2-year college = junior college. ** Based on the Japanese Standard Classification of Occupations. Indented rows under Professional indicate subcategories; their *n* values are included in the total for Professional.

## Data Availability

The de-identified interview data supporting the findings of this study are available from the corresponding author upon reasonable request. The data are not publicly available due to privacy and ethical restrictions.

## Abbreviations

The following abbreviations are used in this manuscript:

NBS	newborn bloodspot screening
stdNBS	standard newborn bloodspot screening
eNBS	expanded newborn bloodspot screening
SMA	spinal muscular atrophy
SCID	severe combined immunodeficiency

## Appendix A. Guide for Semi-Structured Interviews with Parents

*Introduction:*

My name is Y.W./M.N./N.T./F.K. I am a genetic counselor at Shizuoka General Hospital.

*Explanation of the Purpose of the Study:*

The purpose of this interview is to clarify what information parents receive regarding expanded newborn bloodspot screening (eNBS), how they understand that information, how they make their final decisions, and what expectations and concerns they experience during this process.

*Clarification of Key Terminology:*

In this study, eNBS refers to a fee-based blood test conducted primarily by the Shizuoka Prefecture Rare Disease Network. This screening is separate from the long-established standard newborn bloodspot screening (stdNBS) program, which screens for congenital metabolic disorders and other conditions.

*Right to Skip Questions:*

If, for any reason, you do not wish to answer a question, please let me know, and we will move on to the next question.

*Background Questions for Participants:*

What is your age? What is your partner’s age?

What is your occupation? What is your partner’s occupation?

What is your highest level of education? What is your partner’s highest level of education?

How many children do you have? What are your children’s ages?

Who lives with you?

*Interview Questions:*

1. How did you obtain information about eNBS?
2. How well did you understand that information?
3. What process led to your final decision about whether to accept eNBS?
4. What factors influenced your decision?
5. What anxieties or concerns did you have about accepting eNBS?
6. How did you address those anxieties or concerns?
7. *If heredity or genetic testing has not been mentioned:* How do you feel about genetic testing in newborns?
8. What additional information would have been helpful when deciding whether to accept eNBS?
9. What kind of support from healthcare providers would be helpful?
10. What advice would you give to other parents in a similar situation?
